# The Old Churchyard

**Published:** 1883-07

**Authors:** 


### THE OLD CHURCHYARD.

Breathe soft and low, O whispering wind,
Above the tangled grasses deep,
Where those who loved me long ago
Forgot the world and fell asleep.
No towering shaft or sculptured urn,
Or mausoleum's empty pride,
Tells to the curious passer-by
Their virtues, or the time they died.

I count th old familiar names,
O'ergrown with moss and lichen g ay,
Where tangled brier and creeping vine
Across the crumbling tablets stray.
The summer sky is softly blue ;
The birds still sing the sweet, old strain ;
But something from the summer-time
Is gone that will not come again.

So many voices have been hushed—
So many songs have ceased for aye—
So many hands I used to touch
Are folded over hearts of clay.
The mossy world recedes from me—
I cease to hear its praise or blame ;
The mossy marbles echo back
No hollow sound of emp y fame.

I only know that, calm and still,
They sleep beyond life's woe and wail,
Beyond the fleet of sailing clouds,
Beyond the shadow of the vale ;
I only feel that, tired and worn,
I halt upon the highway bare,
And gaze with yearning eyes beyond
To fields that shine supremely fair.

